# Effects of MOVPE Growth Conditions on GaN Layers Doped with Germanium

**DOI:** 10.3390/ma14020354

**Published:** 2021-01-13

**Authors:** Dario Schiavon, Elżbieta Litwin-Staszewska, Rafał Jakieła, Szymon Grzanka, Piotr Perlin

**Affiliations:** 1 Optoelectronic Devices Laboratory, Institute of High Pressure Physics, Polish Academy of Sciences, al. Sokołowska 29/37, 01-142 Warsaw, Poland; szgrzanka@unipress.waw.pl (S.G.); piotr@unipress.waw.pl (P.P.); 2 Laboratory of Nitride Semiconductor Physics, Institute of High Pressure Physics, Polish Academy of Sciences, al. Sokołowska 29/37, 01-142 Warsaw, Poland; ela@unipress.waw.pl; 3 Laboratory of X-ray and Electron Microscopy Research, Institute of Physics, Polish Academy of Sciences, al. Lotników 32/46, 02-668 Warsaw, Poland; jakiela@ifpan.edu.pl

**Keywords:** Ge, germanium, doping, GaN, gallium, nitride, MOVPE, epitaxy

## Abstract

The effect of growth temperature and precursor flow on the doping level and surface morphology of Ge-doped GaN layers was researched. The results show that germanium is more readily incorporated at low temperature, high growth rate and high V/III ratio, thus revealing a similar behavior to what was previously observed for indium. V-pit formation can be blocked at high temperature but also at low V/III ratio, the latter of which however causing step bunching.

## 1. Introduction

In the last few years there has been a renewed interest in highly doped *n*-GaN layers produced by metal-organic vapor-phase epitaxy (MOVPE). Such layers are very important for the optimization of the operative voltage of GaN-based electronic devices, especially those with tunnel junctions [1], as well as for controlling the layers’ refractive index thanks to the plasmonic effect [2]. For all these applications, it is desirable to reach higher doping levels than those achievable with silicon as a donor impurity. In fact, silicon proved inadequate at providing a doping level higher than $10^{19}$ cm${}^{-3}$ because of a general deterioration of the surface morphology [3] probably caused by the antisurfactant property of Si [4]. Recently, germanium has emerged as a better alternative to silicon as a donor dopant. While it has a similar activation energy [5,6] and provides similar carrier mobility at every doping level [7], germanium has proved capable of attaining doping levels beyond $10^{20}$ cm${}^{-3}$ without negatively affecting surface morphology [3,8] nor introducing mechanical strain in the epitaxial structure [3,9]. Such a high doping level is sufficient to bring about a decrease of the refractive index of the material, and opens up possibilities of using GaN layers as waveguide claddings in laser structures in place of strained AlGaN layers [2,10].

One problematic aspect of GaN:Ge (i.e., Ge-doped GaN) growth is that the incorporation efficiency of germanium depends strongly on the MOVPE growth conditions. For example, Fritze et al. [3] reported that their dopant precursor flow for germanium was 2–3 orders of magnitude higher than for silicon at the same resulting doping level. Specifically, for a doping level of $1.9\times 10^{20}$ cm${}^{-3}$, their Ge/Ga precursor ratio was probably as high as 1/3 in the gas phase. However, Kirste et al. [11] obtained a similar Ge-doping level with a Ge/Ga precursor ratio that is two orders of magnitude lower, and therefore quite similar to conventional Si doping. The uncertainty about the incorporation efficiency makes it difficult to control the doping level appropriately, and is therefore an interesting subject of study.

Another challenge of GaN:Ge growth is the difficulty of avoiding the formation of V-pits (hexagonal inverse-pyramidal pits). Such morphological defects have longtime been known to form at low growth temperature, such as in the case of InGaN layers (650–800 °C), on top of screw (or mixed) dislocations [12]. During the growth of GaN:Ge layers, however, these can be produced at temperatures as high as 1075 °C [3], depending possibly also on the concentration of the incorporated germanium. The physical mechanism proposed to explain their formation involves the segregation of liquid germanium droplets on top of screw dislocations. V-pits are then formed as a consequence of the disturbance provoked by the droplets themselves [13,14]. Different strategies have been proposed to clear the surface of V-pits, including reducing the density of screw dislocations [3] or, particularly in the case of hydride vapor-phase epitaxy (HVPE), reducing the partial pressure of diatomic hydrogen in the reactor chamber [13].

Until today, there has not yet been a systematic investigation detailing how the growth conditions affect germanium incorporation and V-pit formation. Advanced modeling of the growth of group III–nitride materials based on density-functional theory (DFT) have generally proved to be adequate methods for understanding temperature and precursor-flow trends in these layered materials [15,16]. This article, however, will focus on presenting direct experimental data. In particular, we will show that germanium incorporation behaves similarly to indium incorporation, suggesting that the underlying physical mechanism is the same. Moreover, we will offer an alternative solution to the issue of V-pit formation based on the deliberate switch to a growth mode that favors step bunching, followed by mechano-chemical polishing (MCP).

## 2. Effect of Temperature and TMGa Flow

A starting series of 10 samples, conveniently labelled with the letters “a”–“j”, were grown at different temperatures and Ga-precursor flows on sapphire substrates by metal-organic vapor-phase epitaxy (MOVPE) in an Aixtron close-coupled-showerhead reactor. For all samples, the growth pressure was kept at 10 kPa, the showerhead gap at 6 mm and the total gas flow into the reactor at 8 slm using H_2_ as carrier gas. The precursor gases are ammonia (NH_3_), trimethylgallium (TMGa) and germane (GeH_4_), the latter being supplied from a mix of 10% GeH_4_ and 90% H_2_ of six-nines purity. For all samples, the NH_3_ flow was kept at 2 slm and the GeH_4_ flow at 7.6 μmol/min, which is almost the minimum allowed by our dedicated mass-flow controller. The epitaxial structure consists of a 5-μm-thick undoped GaN buffer layer, and a GaN:Ge test layer with thickness in the range 0.5–1.2 μm. The growth rate (GR) was monitored by a laser reflectometer and the susceptor surface temperature (*T*) was measured by an Aixtron Argus™ dual-wavelength pyrometer with emissivity correction. The samples were characterized by secondary-ion mass spectrometry (SIMS) and Van-der-Pauw Hall-effect measurements. The results are summarized in Table 1. Note that not all samples were measured by SIMS, and that sample “j” was highly resistive so it could not be characterized by Hall effect. However, wherever both types of data are available, we see that the electron density matches the germanium concentration within the uncertainty of the SIMS measurement, which is in agreement with the general expectation that germanium dopant is mostly activated at room temperature [5,6,17].

**Figure 1:**
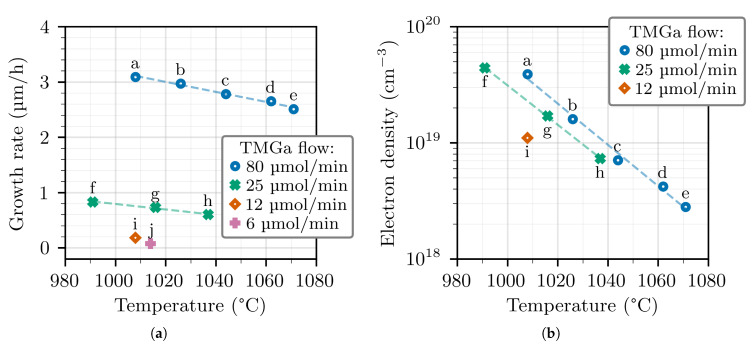
Growth rate (GR) (**a**) and electron density (**b**) for GaN:Ge layers grown at different temperatures and TMGa flows, while the NH${}_{3}$ and GeH${}_{4}$ flows are kept at 2 slm and 7.6 μmol/min, respectively. The dashed lines are linear fits to the data.

Figure 1 shows the GR and the electron density (from Hall measurements) as a function of temperature. The GR depends on the TMGa flow, of course, but it also depends on the temperature. The GR dependence on the temperature is a known phenomenon that is possibly caused by a combination of parasitic reactions and thermal decomposition. In the context of group III–nitride material epitaxy, parasitic reactions are chemical reactions occurring in the gas phase between NH_3_ and the metalorganic precursors, and producing nanoparticles that are quickly carried out of the reactor chamber (while the thermophoretic effect keeps them from reaching the growth surface). Such reactions are especially well documented for the growth of AlGaN layers with TMGa and TMAl precursors [18,19], where especially TMAl is prone to react and form [(CH_3_)_2_AlNH_2_]_n_ oligomers from which the nanoparticles can nucleate [20]. Thermal decomposition, on the other hand, is the reverse reaction of growth itself and is caused by the breaking of the chemical bonds between atoms at the surface of the uppermost layer. It has been observed for GaN layers and it is known to be greatly enhanced by the presence of H_2_ in the gas flow [21,22].

**Figure 2:**
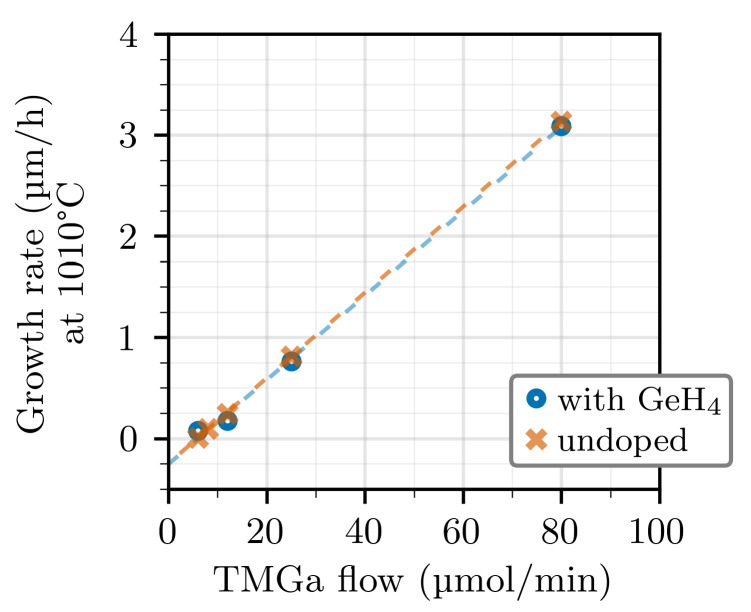
GR of Ge-doped and undoped GaN layers grown at 1010 °C and different TMGa flows. The GR of the doped layers is interpolated from the data of Figure 1a. Note that the data for doped and undoped layers overlap on the same line and that this line crosses the *x*-axis for a TMGa flow of 7 μmol/min.

To better show which mechanism is prevailing in our case, we interpolated the GR corresponding to each TMGa flow at the temperature of 1010 °C. The resulting values are plotted in Figure 2. In addition to the Ge-doped samples, the plot also includes the GR of undoped GaN layers, which were grown in the exact same conditions except that no GeH_4_ was introduced into the reactor. We note that the GR drops to zero for a TMGa flow close to 7 μmol/min, which must be the flow at which the supply of ad-atoms from the precursors matches the loss due to thermal decomposition. This clearly proves that thermal decomposition is taking place. On the other hand, the same data exclude the possibility that parasitic reactions are occurring, because the relation between the GR and the TMGa flow appears to be linear, whereas parasitic reactions would rather result in a drooping curve. We also note that GeH_4_ does not have a significant influence on either the GR or the decomposition rate, because the corresponding lines on the plot do not have different slopes, nor do they have different intercepts with the *x*-axis. We cannot exclude, however, that parasitic reactions could become relevant at higher GeH_4_ flow or higher temperature.

As for Figure 1b, the concentration of germanium atoms in the layers, which we take to be equal to the electron density, is clearly seen to decrease for increasing temperature at each TMGa flow. Moreover, when comparing samples grown with different TMGa flows (but with the same GeH_4_ flow of 7.6 μmol/min), the samples grown with the highest TMGa flow turn out to be those with the highest doping level, which is against our expectations, considering that the Ge/Ga molar ratio in the gas phase is actually smaller. This contrast starkly from the case of silicon, where the doping level is found to be proportional to 1/GR and mostly independent of the temperature [23]. It means that the incorporation efficiency of germanium, unlike silicon, varies as a function of temperature and GR. In fact, our data indicate that the incorporation of germanium behaves similarly to the incorporation of indium in InGaN layers, which is also more easily incorporated at high GR and low temperature [24]. In the case of indium, this is commonly explained by the fact that indium ad-atoms desorb very easily from the growth surface due to the high vapor pressure of indium over (In)GaN [25]. In the case of germanium, given that we have already excluded the possibility of significant parasitic reactions in these growth conditions, we can presume that the mechanism may be the same as it is for indium. At first, the juxtaposition of germanium and indium may seem odd considering that they belong to two different groups in the periodic table. However, germanium and indium have an additional electron shell with respect to silicon and gallium. This results in longer and weaker bonds with the neighboring atoms, and a higher probability to desorb from the growth surface.

## 3. Effect of GeH_4_ and NH_3_ Flow

**Figure 3:**
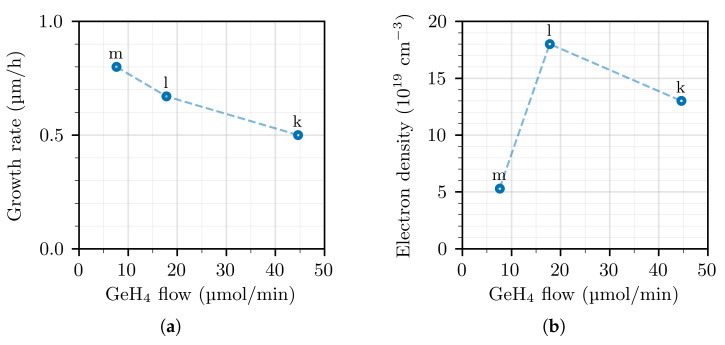
GR (**a**) and electrons density (**b**) for GaN:Ge layers grown at the TMGa flow of 25 μmol/min, as a function of GeH${}_{4}$ flow.

The following series of samples (labelled “k”–“n”, see Table 2) were grown with NH_3_ flow reduced to 0.5 slm from the previous 2 slm. We consider at first samples “k”–“m”, which are grown at approximately the same growth temperature of 970–976 °C but with variable GeH_4_ flow. Their GR and electron density are shown in Figure 3. It is found that the electron density (and therefore the concentration of incorporated germanium) does not depend linearly on the GeH_4_ flow, but instead peaks somewhere at about 15–30 μmol/min and then decreases for increasing GeH_4_ flow. Even this behavior, where the incorporation of a given species saturates and then starts to decrease for increasing precursor flow, has been observed for indium in InGaN layers [26,27]. In the case of indium, Guo et al. [27] have tentatively explained the decreasing trend after the peak as the effect of parasitic reactions. The same could be true for germanium since, even though we demonstrated that no parasitic reactions happen at the GeH_4_ flow of 7.6 μmol/min, we cannot exclude that parasitic reactions could be happening at the considerably higher flow of 45 μmol/min (despite the slightly lower temperature of 970 °C). Assuming that these parasitic reactions involve both GeH_4_ and TMGa as reagents, they can also explain the reduction of GR that is observed for increasing GeH_4_ flows in Figure 3a.

**Figure 4:**
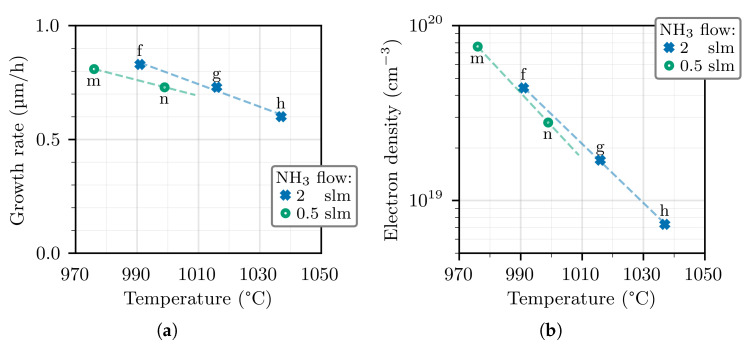
GR (**a**) and electrons density (**b**) in GaN:Ge layers grown at different temperatures and NH${}_{3}$ flows, while the TMGa and GeH${}_{4}$ flows are kept at 26.8 and 7.5 μmol/min, respectively.

It is also useful to compare the samples “f”–“h” from the first series with the samples “m”–“n” from the second series, which are grown with the same TMGa and GeH_4_ flows but different NH_3_ flows. Their GR and electron density is shown in Figure 4. Both the GR and the electron density are slightly lower in the second series with respect to the first series, when comparing at the same temperature. The fact that the incorporation of the more volatile group-III species is enhanced at high V/III ratio has also been confirmed for InGaN growth [27].

## 4. Surface Morphology

**Figure 5:**
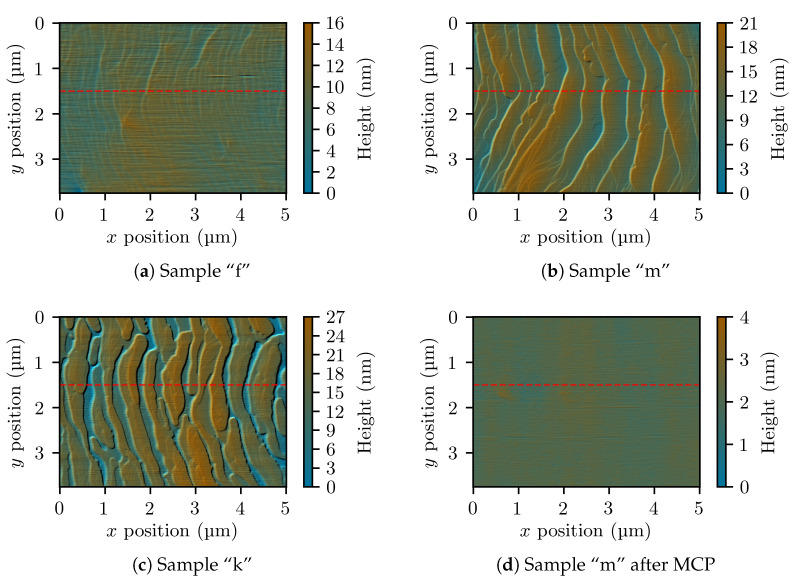
Atomic-force microscopy (AFM) scans of four GaN:Ge layers. Subfigure (**d**) shows the same sample as subfigure (**b**) but after mechano-chemical polishing (MCP). The sections marked by the dashed lines are shown in Figure 6.

A remarkable difference between the first and the second series of samples (grown with a NH_3_ flow of 2 slm and 0.5 slm, respectively) resides in the surface morphology. This was analyzed by optical microscopy and atomic-force microscopy (AFM). The samples of the first series, with the only exception of those grown at temperatures above 1050 °C (i.e., samples “d” and “e”), are characterized by the presence of V-pits with a density of $10^{7}$ cm${}^{-2}$. The size of the V-pits tends to decrease as the temperature approaches 1050 °C, by which they disappear completely. Unfortunately, as was discussed above, it is not possible to incorporate a satisfactory amount of germanium dopant when growing at temperature higher than 1050 °C. Aside of the V-pits, the surface appears otherwise flat, and growth steps are clearly visible in the AFM scans such as the one shown in Figure 5a.

**Figure 6:**
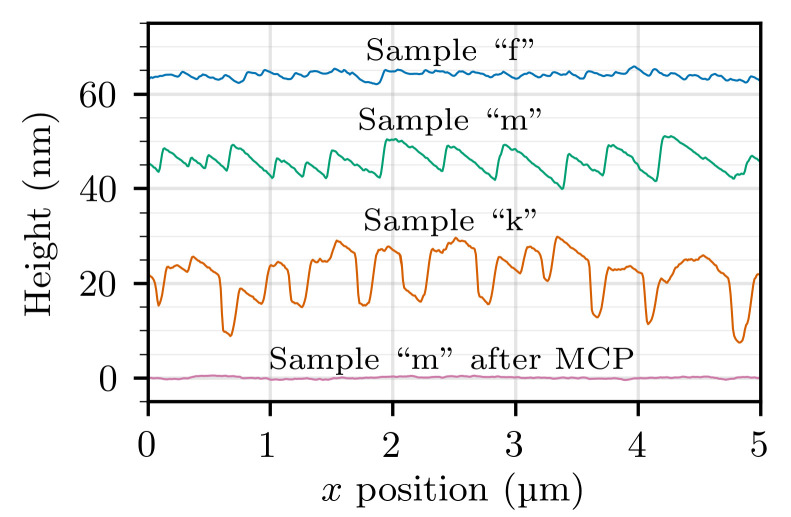
Sections extracted along the dashed lines from the scans shown in Figure 5. The lines have been displaced vertically so that they do not overlap each other.

On the other hand, the samples of the second series are strongly affected by step bunching. Instead of individual atomic steps, fewer but higher macrosteps are observed in AFM scans, as can be seen on sample “m” shown in Figure 5b (for this scan, an area without V-pits was accurately chosen). Moreover, the macrostep height depends on the germanium concentration. Comparing samples “m” and “k” in Figure 5b,c, one can note that the height of the macrosteps increases as the GeH_4_ flow is increased from 7.6 μmol/min to 45 μmol/min. To make a quantitative comparison possible, sections of all the AFM scans in Figure 5 performed along the *x*-axis are shown in Figure 6. It can be observed that the macrostep height increases from 8 nm to 15 nm, corresponding to about 30 and 60 monolayers, respectively. However, no V-pits were found in the samples of the second series. In our experience, the lack of V-pits in samples grown at low temperature is always connected to the emergence of step bunching. Once again, a similar case where the lowering of the NH_3_ flow produced a significant change of the growth morphology was observed in InGaN layers [28].

While step bunching is certainly not a desirable feature by itself, in this case it can prove useful in that it blocks V-pit formation. A good reason to prefer step bunching to V-pits is that the affected layer can be planarized more easily, for example by overgrowing with a high-temperature GaN layer, or by MCP. To prove this, sample “m” was treated with a 10-min-long MCP process, by which we estimate that approximately 100 nm were etched. The AFM scan of the resulting flat surface is shown in Figure 5d. We believe that the same result could likely be obtained with a shorter MCP run as well.

## 5. Conclusions

The effect of growth temperature and precursor flow on germanium incorporation and surface morphology was studied in depth. A total of 14 samples with GaN:Ge layers were grown and characterized. It was found that the germanium incorporation depends on the growth conditions in a similar way to indium incorporation in InGaN layers, namely it increases at low temperature, high growth rate and high V/III ratio, whereas it does not increase linearly with the precursor flow but instead it saturates and then it slowly decreases. The effect of pressure was not tested but, based on the behavior of indium, one can expect that the incorporation of germanium would increase when the pressure is low [29]. Even though our study involved only GaN samples, with caution some conclusions can also be taken for the other group III–nitride alloys. The strong dependence of the germanium incorporation on temperature indicates that germanium may not be the optimal dopant species for Al-rich AlGaN alloys, which are typically grown at high temperature to improve surface ad-atom diffusion [30,31]. On the other hand, if the temperature is decreased to the typical values for InGaN growth (650–800 °C), it is possible that germanium’s tendency to desorb would cease and its incorporation efficiency would become approximately constant, as it is for silicon at the typical growth conditions for GaN (950–1050 °C).

**Figure 7:**
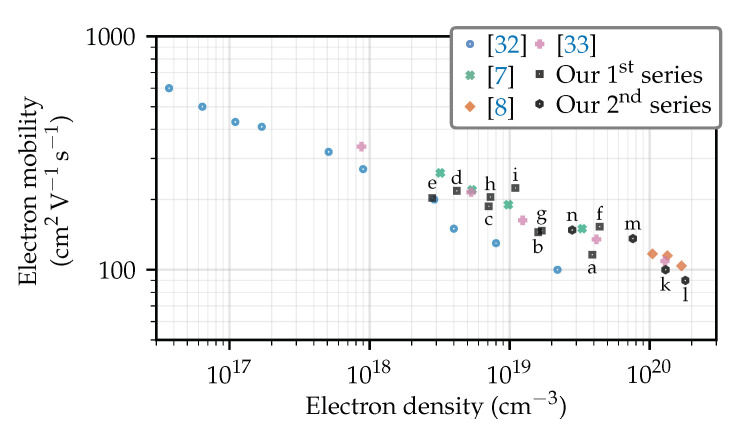
The electron mobility of all samples as a function of the electron density and compared to equivalent values from the literature [7,8,32,33].

The samples of our second series, grown with a reduced NH_3_ flow, were affected by step bunching but were also free of V-pits. Within the first series, instead, only samples “d” and “e” were lacking V-pits, however they also incorporated considerably less germanium because of the high temperature of growth. The macrosteps produced by step bunching are of course undesirable but we showed that they could at least be removed by means of a quick 10-min MCP process. This solution is adequate for structures in which thick GaN:Ge layers are needed, as in the case of the cladding layers of InGaN/GaN laser diodes. As for the electron mobility in our GaN:Ge layers, it is about as high as the other values reported in the literature (see Figure 7), so there can be little doubt as to the quality of our samples.

## Figures and Tables

**Table 1:** Summary of the growth conditions and characterization results of the first series of samples, where only the growth temperature and the TMGa flow were varied. The [Ge] column shows the concentration of germanium impurities in the test layers as measured by SIMS. The *n* and μ columns show the electron density and mobility from the Hall-effect measurements.

Id	*T*	GR	NH${}_{3}$	TMGa	GeH${}_{4}$	[Ge]	*n*	μ
	(°C)	$\left(\frac{\mu m}{h}\right)$	(slm)	$\left(\frac{\mu \mathrm{mol}}{\min}\right)$	$\left(\frac{\mu \mathrm{mol}}{\min}\right)$	$\left({\mathrm{cm}}^{-3}\right)$	$\left({\mathrm{cm}}^{-3}\right)$	$\left(\frac{{\mathrm{cm}}^{2}}{V\;s}\right)$
a	1008	3.09	2	80	07.6	3–$5\times 10^{19}$	$3.9\times 10^{19}$	116
b	1026	2.97	2	80	07.6		$1.6\times 10^{19}$	145
c	1044	2.78	2	80	07.6		$7.1\times 10^{18}$	187
d	1062	2.65	2	80	07.6		$4.2\times 10^{18}$	218
e	1071	2.51	2	80	07.6		$2.8\times 10^{18}$	203
f	0991	0.83	2	25	07.6		$4.4\times 10^{19}$	153
g	1016	0.73	2	25	07.6	1–$2\times 10^{19}$	$1.7\times 10^{19}$	147
h	1037	0.60	2	25	07.6		$7.3\times 10^{18}$	205
i	1008	0.19	2	12	07.6	1–$2\times 10^{19}$	$1.1\times 10^{19}$	224
j	1014	0.05	2	06	07.6	3–$7\times 10^{18}$		

**Table 2:** Summary of the growth conditions and characterization results of the second series of samples, where the NH${}_{3}$ flow is reduced to 0.5 slm. The [Ge] column shows the concentration of germanium impurities in the test layers as measured by secondary-ion mass spectrometry (SIMS). The *n* and μ columns show the electron density and mobility from the Hall-effect measurements.

Id	*T*	GR	NH${}_{3}$	TMGa	GeH${}_{4}$	[Ge]	*n*	μ
	(°C)	$\left(\frac{\mu m}{h}\right)$	(slm)	$\left(\frac{\mu \mathrm{mol}}{\min}\right)$	$\left(\frac{\mu \mathrm{mol}}{\min}\right)$	$\left({\mathrm{cm}}^{-3}\right)$	$\left({\mathrm{cm}}^{-3}\right)$	$\left(\frac{{\mathrm{cm}}^{2}}{V\;s}\right)$
k	0970	0.50	0.5	25	45.0		$1.3\times 10^{20}$	100
l	0970	0.67	0.5	25	18.0		$1.8\times 10^{20}$	090
m	0976	0.81	0.5	25	07.6	6–$10\times 10^{19}$	$7.6\times 10^{19}$	136
n	0999	0.73	0.5	25	07.6		$2.8\times 10^{19}$	148

## Data Availability

Data is contained within the article.
